# Direct Formation and Structural Characterization of Electride C12A7

**DOI:** 10.3390/ma12010084

**Published:** 2018-12-27

**Authors:** J.R. Salasin, S.E.A. Schwerzler, R. Mukherjee, D.J. Keffer, K.E. Sickafus, C.J. Rawn

**Affiliations:** 1 Department of Materials Science and Engineering, University of Tennessee, Knoxville, TN 37996 USA; jsalasin@vols.utk.edu (J.R.S.); sschwerz@vols.utk.edu (S.E.A.S.); dkeffer@utk.edu (D.J.K.); kurt@utk.edu (K.E.S.); 2 Center for Materials Processing, University of Tennessee, Knoxville, TN 37996, USA; 3 Department of Physics, Lovely Professional University, Phagwara 144401; India; rupam.23644@lpu.co.in

**Keywords:** direct electride synthesis, C12A7, Ca_12_Al_14_O_33_, mayenite, electride structure, electride

## Abstract

Ca_12_Al_14_O_33_ (C12A7 or Mayenite) is a material whose caged clathrate structure and occluded anionic species leads to significant functionality. The creation of occluded anionic vacancies leads to the injection of localized electrons at the center of the cage, converting the wide band gap insulator to a semi- or metallic conducting material. The conversion to the electride historically requires the synthesis of oxy-C12A7, consolidation, and then reduction to introduce anionic vacancies. This report develops and characterizes an electride formation procedure from three starting points: unconsolidated oxy-C12A7, heterogenous solid-state reactants (CaCO_3_ and Al_2_O_3_), and homogenous non-carbonaceous polymer assisted sol-gel reactants. Electride-C12A7 formation is observed in a vacuum furnace where the reactants are in direct contact with a carbon source. Process time and temperature-dependent structural characterization provides insight into the source of high temperature C12A7 stability, the mechanism of anionic vacancy formation, and the magnitude of ultimate conductivity that cannot be explained by current reduction theories. A new theory is presented where mixed O- and C-occupied cages lead to high temperature stability, oxidation of C species creates anionic vacancies, and an equilibrium between the reducing power of the electride-C12A7 and of the C species leads to the ultimate conductivity achieved by the process. This represents a shift in understanding of the carbonaceous reduction process and the first report of high purity electride-C12A7 formation from heterogenous solid-state reactants and homogenous non-carbonaceous polymer assisted sol-gel reactants.

## 1. Introduction

The mineral mayenite, also known as C12A7 and chemically Ca_12_Al_14_O_33_, has gained attention in the last two decades due to the high degree of functionality afforded by the crystal structure. C12A7 crystallizes in the highly symmetric $I\bar{4}3d\;\left(\mathrm{no}.\;220\right)$ space group with *a* = ≈11.98 Å and two formula units per unit cell resulting in 118 atoms within the unit cell. The functionality of the material results from the clathrate structure where the unit cell is made up of a positively charged framework of twelve interconnected cages and occluded stabilizing anions. These anions within the cage are weakly bound to the framework leading to a [framework]: occluded-anion notation, ${\left[{\mathrm{Ca}}_{24}{\mathrm{Al}}_{28}O_{64}\right]}^{4+}:2O^{2-}$. Manipulating cationic doping of the framework and mobile occluded anions in C12A7 leads to potential applications in inorganic and organic synthesis; surface treatments; catalytic reactions; as a high purity and high density anionic source; transparent conductive oxide; direct writable transparent wires and media; luminescent for displays, lighting, or photoelectric devices; gas and biomass reforming; and even an antibacterial agent among numerous other applications; Liao et al. present a comprehensive review on the potential applications of the stoichiometric ${\left[{\mathrm{Ca}}_{24}{\mathrm{Al}}_{28}O_{64}\right]}^{4+}:2O^{2-}$ as well as its derivatives [1].

Hosono et al. have conducted extensive research on C12A7 most notably on the formation of the ${\left[{\mathrm{Ca}}_{24}{\mathrm{Al}}_{28}O_{64}\right]}^{4+}:e_{\left(2*\partial \right)}^{-}O_{\left(1-\partial \right)}^{2-}$ derivative [2,3]. The stabilizing $O^{2-}$ anion is replaced by electrons that are localized in the “potential well” created by the cationic cage leading to the classification of an electride. C12A7 was the first room-temperature stable inorganic electride bringing the realization and application of electrides to ambient conditions [4]. The formation of the electride seems to be robust with many different formation routes, all focusing on processing C12A7 in the presence of a preferentially oxidized sacrificial target, known in this context as an “oxygen getter” [5]. The most practical methods involve utilizing carbonaceous environments due to experimental ease and lack of post processing of previously synthesized and consolidated samples. Kim and Hosono have published a review on the characteristics of the electride C12A7 [3].

**Figure 1:**
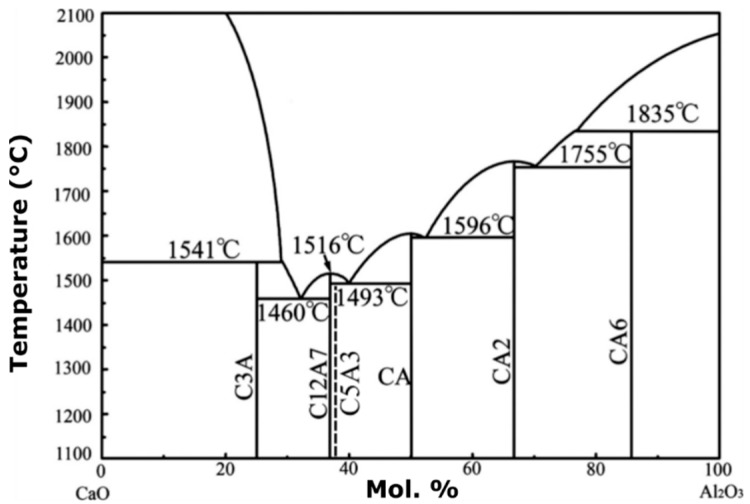
Calculated CaO–Al_2_O_3_ phase diagram under ambient “humid” synthesis conditions, modified from Liao et al. [1]. The metastable C5A3 position is represented by the dotted line; however, C5A3 is only observed to form under non-humid conditions.

The stoichiometric compound, ${\left[{\mathrm{Ca}}_{24}{\mathrm{Al}}_{28}O_{64}\right]}^{4+}:2O^{2-}$ is an invariant binary compound in the CaO–Al_2_O_3_ system at approximately 37 mol% ${\mathrm{Al}}_{2}O_{3}$ (Figure 1). In the shorthand notation in Figure 1, C represents CaO, while A refers to the sesquioxide Al_2_O_3_. Gfellar and Salasin and Rawn have published reviews on the structure of C12A7 and electride C12A7 [5,6]. On either side of the invariant C12A7 is a two-phase region containing C12A7 and two other invariant binary compounds ${\mathrm{Ca}}_{3}{\mathrm{Al}}_{2}O_{6}$ (C3A) and ${\mathrm{CaAl}}_{2}O_{4}$ (CA). The compound ${\mathrm{Ca}}_{5}{\mathrm{Al}}_{6}O_{14}$ (C5A3) is meta-stable and is frequently discussed in the literature when dealing with the C12A7 system. It is interesting to note that the shorthand notation provides a convenient means to write mass balance reactions for the formations of calcium aluminate compounds found in the binary CaO–Al_2_O_3_ phase diagram. 

**Figure 2:**
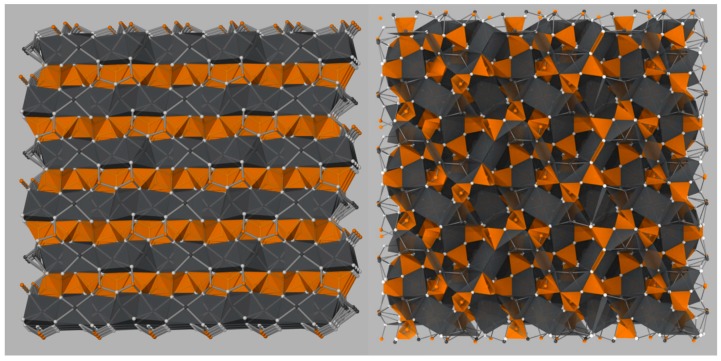
Comparison of the ordered layered C5A3 structure (**left**) and the C12A7 cage structure (**right**). Ca cations and coordination polyhedra are shown in gray, Al cations and coordination polyhedra are shown in orange, and oxygen anions are shown in white. Reproduced from Salasin and Rawn [5].

Thermodynamic solid-state synthesis (SSS) investigations into the formation of C12A7 from ${\mathrm{CaCO}}_{3}$ and ${\mathrm{Al}}_{2}O_{3}$ found that the first phases to crystalize are C5A3 and C3A [7,8]. The C5A3 concentration increases until approximately 950 °C, after which C5A3 is consumed in favor of the formation of C12A7. C5A3 and C12A7 are close in stoichiometry, $7{\mathrm{Ca}}_{5}{\mathrm{Al}}_{6}O_{14}+\mathrm{CaO}\to 3{\mathrm{Ca}}_{12}{\mathrm{Al}}_{14}O_{33}$, and crystal structure. Both structures contain octahedrally coordinated Ca and tetrahedrally coordinated Al cations, but C5A3 is an ordered layered structure, while C12A7 is a disordered clathrate structure (Figure 2). As the temperature is increased, the formation of CA occurs until approximately 1200 °C when C3A and CA combine to form C12A7 [7]. These observations suggest there are two formation pathways from solid-state reactants: (1) C5A3 + C3A at low temperatures (<900 °C) when cationic species are well-mixed [9,10], and (2) C3A + CA at high temperatures (>1100 °C), observed when long diffusion lengths of reactants are present [7,8]. Recent in situ kinetic studies suggest that different pathways might occur with C12A7 being a kinetically favorable phase to form directly at lower temperature and C5A3 only observed as a decomposition product of C12A7 [11].

The relationship between C3A, CA, and C5A3 is further elucidated when investigating the decomposition of C12A7. The presence of occluded anions is heavily correlated to the stability of the clathrate structure, and in the absence of any template anions, i.e., under dry and/or reducing conditions, C12A7 is not thermodynamically favorable and decomposes. This was first observed in single crystal growth experiments where moisture or oxygen was needed to nucleate the C12A7 framework [3]. The decomposition products were reported to vary based on process temperature and were either C5A3 + C3A or C3A + CA; a historical summary of formation products in various oxidizing, reducing, inert, dry, and hydrated atmospheres has been compiled by Kim et al. [3,12]. As the breadth of electride formation research grows, the decomposition of C12A7 continues to limit electride formation at elevated processing temperatures for long process durations. Palacios et al. observed the electride formation and subsequent decomposition of electride-C12A7 powder to C5A3 and C3A through in situ neutron diffraction in a V sample holder heated to 1100 °C under vacuum [13]. Ali et al. observed C12A7 decomposition during electride formation of float zone (FZ) single crystals sealed in an evacuated quartz ampule with Ti powder and fired at 1200 °C for 48 h [14]. Eufinger et al. conducted a study to identify the diffusion of various anions in the C12A7 structure and discovered a correlation between humidity and decomposition of the C12A7 framework [15]. A decomposition analysis found that under dry conditions, C12A7 decomposes at temperatures above 1050 °C, which is correlated with the critical mobility limit for Ca ions [15]. This decomposition was observed in both inert and oxidative atmospheres, where decomposition times were greater than 24 h. The decomposition products of C3A + C5A3 or CA + C3A were observed and correlated with the temperature and atmosphere conditions during the initial temperature ramp [15]. Decomposition was found to nucleate at the macroscopic crystal defects, which agrees well with the calculated Avrami exponent by Palacios et al., suggesting that the limiting rate step of decomposition is the growth of the decomposed products around the C12A7 phase and not the random nucleation of C5A3 products [13,15]. These decomposition times and temperatures agree with those observed in the reduction experiments, but the vacuum and reducing environments may lead to an increase in the rate of the decomposition reaction. Just as in the formation of C12A7, there are two decomposition pathways: (1) C5A3 + C3A, and (2) C3A + CA.

Conversion to ${\left[{\mathrm{Ca}}_{24}{\mathrm{Al}}_{28}O_{64}\right]}^{4+}:e_{\left(2*\partial \right)}^{-}O_{\left(2-\partial \right)}^{2-}$ occurs primarily via the reaction of the occluded oxygen with an oxygen getter [5]. As the electride formation occurs, sample color transitions from white, through green, to black, and the electrical conductivity transitions through an insulating, semi-conducting, and metallic conducting regime, respectively [3]. One oxygen getter reduction method involves processing the synthesized C12A7 powder, single crystal, thin film, consolidated sample, etc., in a C crucible under flowing inert gas. This generates a strong reducing atmosphere in the crucible leading to the proposed reduction of the sample via the following reaction $O_{\left(cage\right)}^{2-}+CO_{\left(g\right)}\to CO_{2\left(g\right)}+2e_{\left(cage\right)}^{-}$ [16]. This is favorable leading, to a conversion with no post processing, but the degree of reduction attainable is not well-reported or discussed and it appears that only limited conversion of $O^{2-}$ to $e^{-}$ occurs. The other proposed C based reduction method involves the replacement of the occluded $O^{2-}$ with $C_{2}^{2-}$ which has a similar valence and ionic radii with 1.4 and 1.2 Å, respectively [17,18]. The carbide anion is then hypothesized to be instantaneously unstable or unstable during cooling and decomposes to either solid C or CO gas creating anionic vacancies and forming the electride. The pathway to anionic exchange is not clear and appears to not be an exchange but a full recrystallization of the decomposition products to C12A7, indicating that decomposition of ${\left[{\mathrm{Ca}}_{24}{\mathrm{Al}}_{28}O_{64}\right]}^{4+}:2O^{2-}$ needs to occur before ${\left[{\mathrm{Ca}}_{24}{\mathrm{Al}}_{28}O_{64}\right]}^{4+}:2C_{2}^{2-}$ formation and electride conversion can occur [17]. The realization of a full CA recrystallization process yields promise for direct formation of electride-C12A7 from reactants. The direct formation of electride-C12A7 from C3A + CA reactant mixtures has been presented as well as from a carbon-rich Pechini sol-gel precursor [17,19,20]. These studies show a clear plateau in electrical conductivity but little theory for this plateau in electronic properties or characterization of the underlying atomic structure has been presented.

This report seeks to study the processing–structure–property relationships of the conversion of oxy-C12A7 to electride-C12A7, as well as the direct formation of electride-C12A7 from both conventional solid-state (CaCO_3_ + Al_2_O_3_) and non-carbonaceous polymer-assisted sol-gel reactants. The polymer-assisted sol-gel reactant mixture benefits from short diffusion pathways achieved in the Pechini synthesis, previously implemented by Khan et al. to create a carbon-C12A7 electride composite, but instead utilizes the environmental carbon instead of reactant carbon to create a consolidated C12A7 sample with low carbon content [20]. The experiments reported here make use of a carbonaceous environment within a high vacuum furnace that results in a carbonaceous dry (low $P_{H_{2}O}$) low-pressure (low $P_{O_{2}}$) environment. Temperature and time are controlled to investigate the formation, stability, and degree of reduction obtainable by the carbonaceous reduction processes. The changes in electronic and atomic structure for the conversion from oxy-C12A7 to electride-C12A7 and direct formation of electride-C12A7 from heterogenous and homogenous reactants can be analyzed post-processing: X-ray diffraction (XRD) is used to characterize the change in the atomic structure, scanning electron microscopy (SEM) is used to evaluate microstructural changes, and electrical resistivity measurements associate the atomic and microstructural features to electronic properties determining the degree of reduction. The results of this characterization are then used to develop a reduction model involving the formation of a mixed C- and O-occupied C12A7 caged structure; C is sourced from the outward solid-state diffusion of C from the sample holder and O is sourced from the extra oxygen present in the solid reactants. The O is explicitly not sourced from any gas phase reactant.

## 2. Materials and Methods

### 2.1. Starting Sample Formulation

The formation of the electride C12A7 was characterized through three methods: a conversion of as-synthesized oxy-C12A7 to electride-C12A7, direct formation of electride-C12A7 from solid-state reactants CaCO_3_ and Al_2_O_3_, and direct formation of electride-C12A7 from non-carbonaceous polymer-assisted sol-gel reactants. The variation in starting points were implemented to characterize the process and elucidate electride formation mechanics. 

For conversion of oxy-C12A7 to electride-C12A7, oxy-C12A7 was first synthesized using solid-state techniques. ${\mathrm{CaCO}}_{3}$ and ${\mathrm{Al}}_{2}O_{3}$ were dried in a vacuum oven at 200 °C and 37 torr for 24 h and then stoichiometric amounts of powders were weighed and homogenized in an alumina milling jar with 10 mm diameter polytetrafluoroethylene (PTFE) milling beads in a vibratory mill. The powder was then pressed into 30 mm pellets with 58 MPa of uniaxial pressure and fired at 1250 °C for 24 h under ambient conditions. Phase equilibria was analyzed with XRD and subsequent firing steps were performed until single phase C12A7 was obtained. No efforts were taken to avoid moisture uptake during synthesis and it is likely the oxy-C12A7 phase formed with occluded $O^{2-}$and $OH^{-}$ stabilizing anions. This is not an issue as the dehydration of oxy-C127 occurs during heating [15,21].

For the direct formation of the electride from heterogenous solid-state reactants, CaCO_3_ and Al_2_O_3_ were used as the starting materials. Stoichiometric amounts of CaCO_3_ and Al_2_O_3_ were mixed in a vibratory mill and used as-mixed for direct electride formation. No calcination or particle size reduction was performed prior to processing in the vacuum environment.

For direct formation of electride-C12A7 from a homogenous reactant mixture, a polymer assisted sol-gel route was implemented; the polymer assisted method was chosen over the conventional amorphous citrate or Pechini method for better control over reactant homogeneity and carbon content. Poly vinyl alcohol (PVA) was used in this synthesis and a 4:1 cation to PVA ratio was chosen. The PVA (molecular mass of 20,000–30,000) was dissolved in deionized water and allowed to age for 24 h. Ca(NO_3_)_2_·4H_2_O and Al(NO_3_)_3_·9H_2_O were used as cation sources and were measured from 1 molar stock solutions whose molarities were quantified through inductively coupled plasma (ICP) spectroscopy. Stoichiometric amounts of nitrate solutions were combined with the PVA solution under vigorous stirring. The solution was aged for 1 h before solvent evaporation was performed at a solution temperature of 90 °C. Near the end of solvent evaporation, the solution exhibited vigorous bubbling before forming a viscous gel; the bubbling relates to the decomposition of nitrate species. The gel was further heated on the hotplate until completely dried. This powder was then ground in a yttria-stabilized zirconia mortar and pestle and calcined to 600 °C and immediately quenched. This ensured the oxidation of all carbonaceous residue and the obtained white powder was used as the starting homogeneous reactant mixture for direct electride formation. 

There were three distinct starting points for electride formation.

1. Pre-synthesized oxy-C12A7 to electride-C12A7 following traditional methods.
2. Heterogenous multi-phase CaCO_3_ and Al_2_O_3_ powder mixture.
3. Homogenous amorphous low-carbonaceous reactant powder.

### 2.2. Processing to Obtain Electride-C12A7 

**Figure 3:**
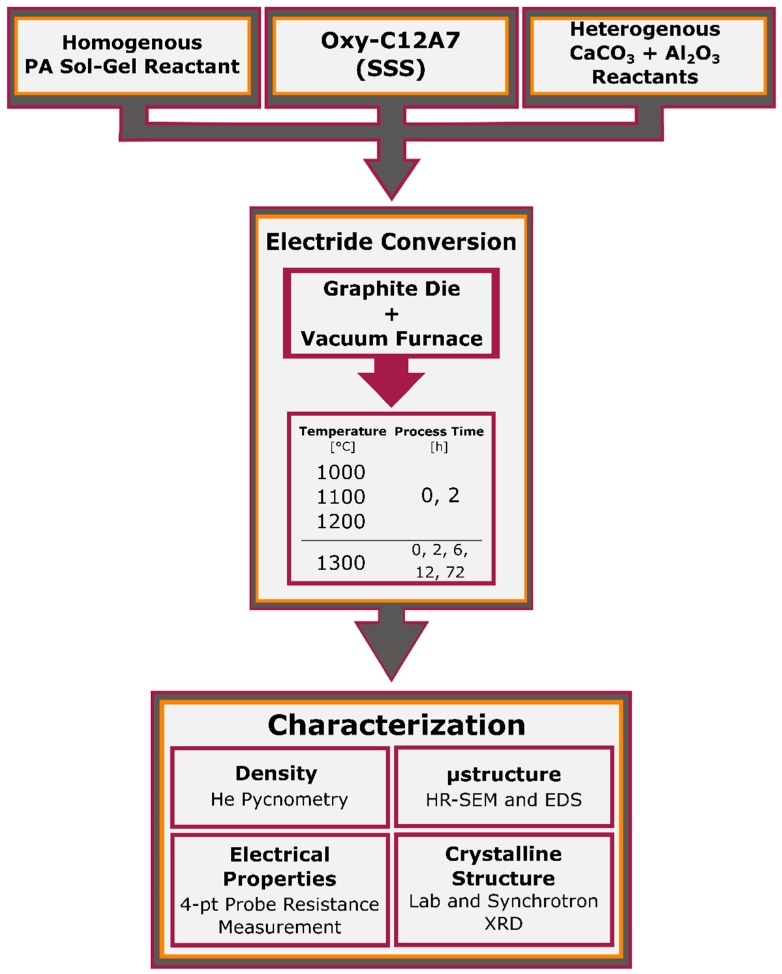
Experimental flow chart for the synthesis, consolidation, and electride formation, and characterization.

**Figure 4:**
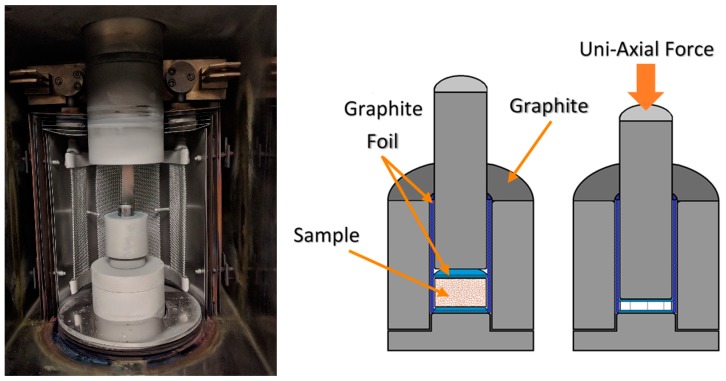
Vacuum furnace and graphite die.

The formation of electride C12A7, in a carbonaceous environment within a vacuum furnace, was performed from the three starting points according to the process flow summarized in Figure 3. The sample chamber was contained within a stainless-steel vacuum furnace with W heating elements and Mo and W shielding. Powder (1 g) was loaded into a 13 mm graphite die placed in the furnace (Figure 4). A graphitic die was used due to the ability to have a carbon source in direct contact with the entire sample; no pressure was applied during the processing. The sample chamber was under high vacuum, 9.0 × 10^−6^ torr, leading to a low oxygen and moisture partial pressure. The temperature of the furnace was raised at a rate of 8 °C/min to the target processing temperature of 1000, 1100, 1200, or 1300 °C. Each sample was only processed to a single temperature and multiple samples were used to evaluate behavior at all temperatures and processing times. The effects of process duration were investigated via increasing the dwell time at the target temperature. The samples’ furnace cooled under vacuum and were prepared for characterization by removing residual surface graphite with a diamond grinding disk and mineral oil. After processing, the samples underwent a full set of characterization to analyze the crystal structure, microstructure, and electrical properties. 

### 2.3. Post-Processing Characterization 

XRD data were collected at the Joint Institute for Advanced Materials (JIAM) Diffraction Facility using a PANalytical Empyrean diffractometer (Malvern Panalytical Ltd., United Kingdom) in Bragg-Brentano geometry with the reflection spinner stage and the PIXcel^3D^ area detector in scanning line mode. The instrumental collimation utilized a 0.125° divergence slit, 0.25° anti-scatter slit, 0.02 rad soller slit, and 10 mm mask on the incident beam side and a 0.125° anti-scatter slit, 0.02 rad soller slit, and Ni-ß filter on the diffracted beam side. Data were collected from 15° to 120° 2*θ* with a step size of 0.00655°, and each step had a 53 second counting time. XRD data were analyzed via the Rietveld method using the GSASII software package [22]. 

Refined parameters included the lattice parameter, sample surface displacement, scale, crystallite size, strain, atomic coordinates, and isotropic thermal parameters; instrumental broadening was characterized through analysis using NIST SRM 640e (Si) (National Institute of Standards and Technology, Gaithersburg, MD, USA). Structural changes as a function of processing parameters were studied; due to the high disorder resulting from various different cage types and heavy correlation between split positions, the thermal parameters were refined isotropically and no attempts were made to refine the fractional occupancies of split positions beyond the basic Ca1 and Ca2 split position [5,6]. To gain better resolution of the split positions and occluded position density, select samples were characterized at the powder diffractometer BM-11 at the Advanced Photon Source (APS). Rietveld refinements were performed using the synchrotron data, and with the increasing probed d-space and resolution, Fourier difference maps were generated along the XY plane at z = 0.25. This plane contains the S_4_ symmetry axis at the cage center allowing for observation of the scattering density at the cage center and Ca split positions to elucidate these heavily correlated low occupancy split positions and occluded position. 

Microstructural characterization was performed to identify the presence of carbon inclusions due to the electride formation technique and the degree of consolidation; consolidated samples were fractured to provide a defect rich and sample post-processing independent microstructure for characterization. Characterization of the microstructure was performed via SEM on either a Zeiss EVO MA15 or a Zeiss Auriga 40 scanning electron microscope (Zeiss, Oberkochen, Germany) using a backscattered detector. Energy dispersive X-ray spectroscopy (EDXS) was performed utilizing a Bruker XFlash 6130 detector for elemental analysis. A Quantachrome Ultrapyc 1200e (Quantachrome, Boynton Beach, FL, USA) was used to determine the density of the consolidated samples via He pycnometry. 

Room temperature resistivity measurements were performed on pellets with >95% theoretical density that were processed for various process times and temperature. The four-point contact technique was used to accurately determine the resistance of the samples. High purity silver paste and 25 µm diameter gold wires were used for electrical contacts. A Keithley 2450 source meter (Tektronix, Beaverton, OR, USA) was used to inject current and sense the potential drop across the sample. Low temperature resistivity measurements were performed on a Quantum Design Physical Property Measure System (PPMS) (Quantum Design Inc., San Diego, CA, USA) at a pressure of 4 Torr in alternative current (AC) mode. All measurements were repeated twice under the same condition in order to confirm the reproducibility. 

## 3. Results

### 3.1. Conversion of Oxy-C12A7 to Electride-C12A7

**Figure 5:**
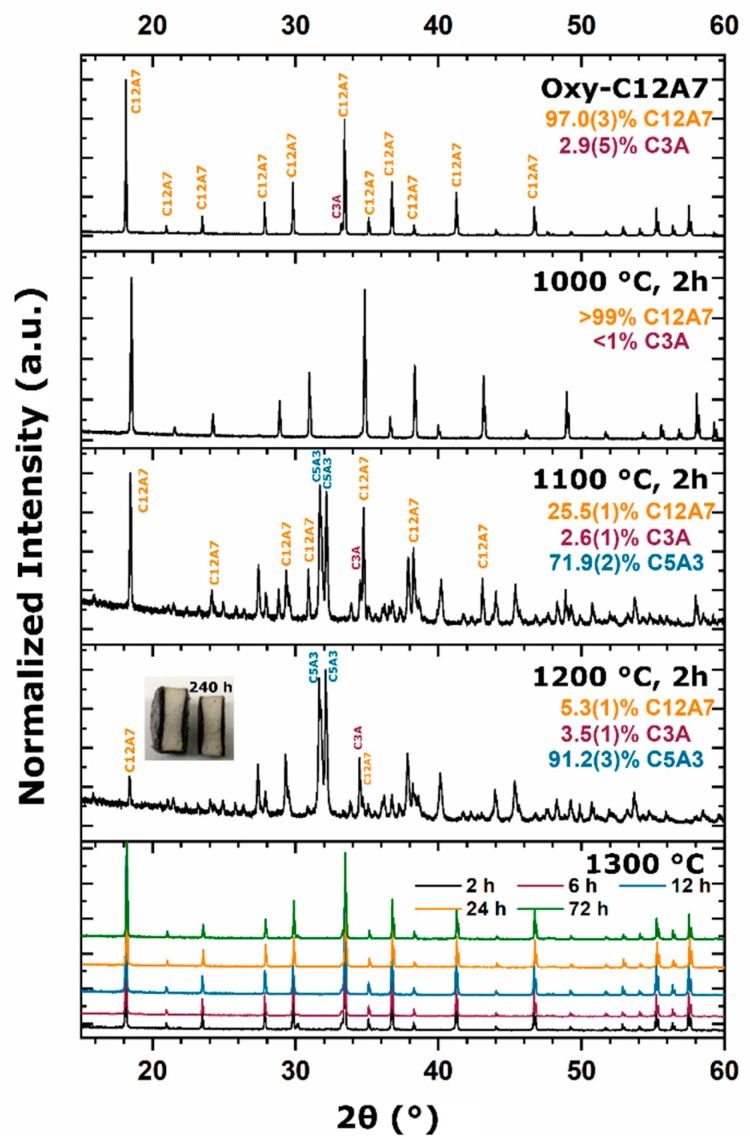
XRD data plotted as a function of processing temperature and at 1300 °C as a function of time. Refined phase fractions are inset on the XRD patterns. Main C12A7 and secondary phase peaks are identified.

Oxy-C12A7 samples processed under high vacuum in the graphite die remained white through 1200 °C with a processing time of 2 h. Laboratory XRD revealed that the C12A7 structure was retained through 1000 °C but C12A7 decomposition to C5A3 and C3A was observed at 1100 and 1200 °C. A cross section of a sample processed for an extend period of 240 h at 1200 °C revealed a clear boundary between black and white regions (inset of Figure 5). XRD characterization identified the black region located near the carbonaceous foil as C12A7 and the white core as C5A3 and C3A, suggesting a diffusion of carbonaceous species into the bulk. When the processing temperature was raised above 1300 °C, a bulk color change through green to black was observed for increasing process times, but when ground to a powder, only samples with a processing time greater than 6 h were green while those processed for less than 2 h were white/manila. When processing time was below 2 h at 1300 °C, XRD data showed the main phase was C12A7 with a few wt% of the C3A and CA secondary phases, suggesting non-equilibrium. After a processing time of 6 h, single phase C12A7 was observed. No C5A3 was observed for any processing time at a processing temperature of 1300 °C.

**Figure 6:**
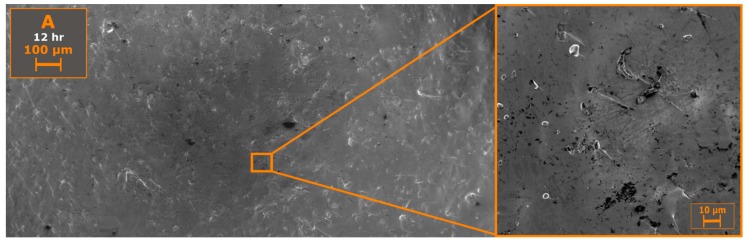
SEM backscattered micrograph on a fractured surface showing low porosity, as well as the presence of carbon nodule impurities.

SEM microstructural characterization, shown in Figure 6, on a fractured surface revealed a high concentration of carbonaceous precipitates, where both large (10 µm) and small nano-sized (<1 µm) C precipitates were observed. Similar to melt grown C12A7 electrides, following the procedure of Kim et al., showing a high concentration of carbonaceous precipitates, suggesting that these precipitates are characteristic of processing at high temperatures in carbonaceous environments [17].

**Figure 7:**
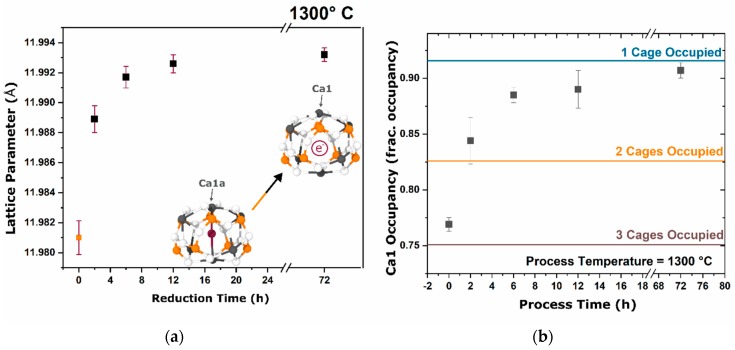
(**a**) Refined lattice parameter of oxy-C12A7 reduced at 1300 °C for various process duration times. The lattice parameter continued to increase as processing time was increased. Inset shows how the contracted occupied cages expanded and the cage dimension increased when cages lose their occluded molecular species, resulting in an increased lattice parameter. Error bars are reported as 3σ. (**b**) Refined Ca1 (unoccupied cage) site occupation. Error bars are reported as σ.

Structural changes between the as-synthesized oxy-C12A7 and carbonaceous process formed electride-C12A7 were quantitatively analyzed using laboratory and synchrotron XRD data. As the oxy-C12A7 changed to the electride-C12A7, a relaxation of the occupied cage shape toward the unoccupied cage shape was observed due to a weaker interaction between the occluded electrons and the framework. Traditionally, this averages out to a predictable increase in lattice parameter to approximately 12.01(2) Å in the fully electron-injected ${\left[{\mathrm{Ca}}_{24}{\mathrm{Al}}_{28}O_{64}\right]}^{4+}:e_{\left(4\right)}^{-}$ [23,24,25]. An increase in lattice parameter was observed at 1300 °C for increasing process durations. The increase in lattice parameter slowed as process duration increased approaching an asymptotic value of approximately 11.9930(6) Å, as shown in Figure 7. 

The system was heavily disordered due to the local changes in cage shape with interactions between the framework and the occluded species. This led to split positions of most atomic positions, most notably the Ca cations, due to the difference in shape of occupied cages, their nearest neighbor cages, and unoccupied cages [26]. With the resolution available in laboratory powder diffraction, the split positions were difficult to resolve. All structural refinements used a modified Boysen structure with only a single split Ca (Ca1a) position [27,28,29]. The actual split cation atomic positions of $O^{2-}$-, $OH^{-}$-, and $e^{-}$-occupied cages have been characterized by Palacios et al. and Sakakura et al. through structural analysis of single crystals [26,30]. Structural characterization through Rietveld refinement identified a decrease in the occupancy of the Ca1a position as process time at 1300 °C was decreased, leading to an increase in the occupancy of the Ca1 position; this indicated an increase in the number of unoccupied cages and a decrease in the number of anion occupied cages. Occupancy of the Ca1 position, associated with an unoccupied cage, increased to an asymptotic value correlated to the occupancy of a single cage per unit cell from an original occupancy correlated to three occupied cages, Figure 7B. This suggested a mixed $O^{2-}$- and $OH^{-}$-occupied cage structures.

**Figure 8:**
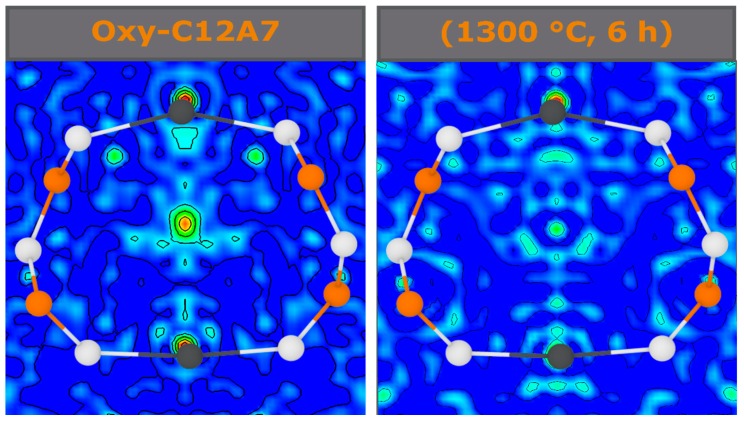
Fourier difference maps of the xy-plane at z = 0.25, slicing through the middle of a cage, generated from the synchrotron diffraction data. Brighter colors indicated an increase in scattering density with both Fourier maps having the same arbitrary scale. A comparison of as-synthesized oxy-C12A7 (**left**) and after processing for 6 h at 1300 °C (**right**). The contour lines are of consistent separation across samples.

This change in the atomic positions and occupancy provides insight into the effect the occluded anion has on the cage. Fourier difference maps generated without an occluded position in the structural model can be used to qualitatively assess the sites of occluded anions, which changes based on anionic type, as well as give an idea for the overall scattering density of those sites. In the as-synthesized powder, the occluded position showed a high circular density at the center of the cage (0.375, 0, 0.25), as well as two smaller areas of density on each side, (0.335, 0.04, 0.25) and (0.35, 0.07, 0.25) corresponding to $OH^{-}$, $O_{2}^{2-}$, and $O^{-}$ species (Figure 8). After processing at 1300 °C for 6 h, the intensity was drastically reduced and in addition to a small amount of density in the center (0.375, 0, 0.25), and equally intense positions were observed on an angle to either side whose maxima lie slightly out of the present plane at the coordinates (0.33779, 0.04639, 0.24038) and (0.33779, −0.04637, 0.25962). The distance between the center atom and the off-center maxima were approximately 0.7 Å. 

**Figure 9:**
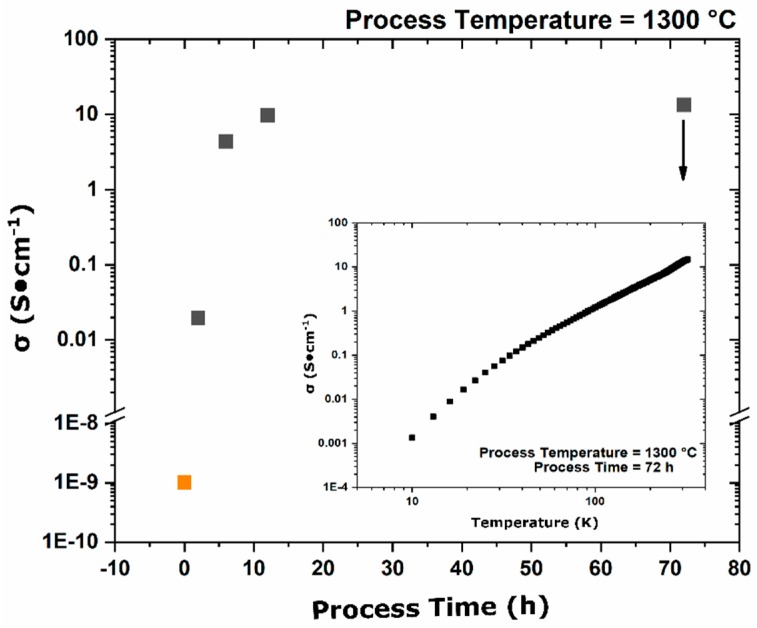
Electrical conductivity of oxy-C12A7 samples processed at 1300 °C for various durations. The conductivity converged to a value of 15 S·cm^−1^. The inset shows the temperature dependence of the sample, which was reduced for 72 h, indicating the sample was semi-conducting.

In processes in which complete conversion to the electride was observed the initial wideband gap insulator transitions through a semi-conducting state ultimately to a metallic temperature invariant conductivity state as the conversion process proceeds. Characterization of the magnitude of conductivity and the proportionality of conductivity as a function of temperature indicated what degree of reduction had been achieved; semi-conducting variable range hopping is an activated process resulting in a change in conductivity as a function of temperature, while metallic type band conductivity is relatively temperature-invariant. Physical property measurements showed an increase in conductivity with the process duration ultimately approaching an asymptotic value of approximately 15 S·cm^−1^ (Figure 9). The temperature dependence of conductivity in the 72-h sample (Figure 9 inset) showed a decrease in conductivity with decreasing temperature, indicating a temperature-activated conductivity mode consistent with variable range-hopping semi-conducting behavior; metallic conductivity was not achieved as the process duration was increased as observed in metal reduction processes. 

A subsequent experiment was performed by processing samples at 1200 °C for 2 h before raising the temperature to 1300 °C for 2 h. The intent was to decompose the C12A7 framework completely to C5A3 and C3A and then raise the temperature and analyze the crystalline product. XRD characterization of the processed sample showed single phase C12A7, compared to a multi-phase (C12A7 + C3A + CA) sample when processed directly at 1300 °C for 2 h (Figure S1). Further, the lattice parameter of 11.9912(2) was larger than the lattice parameter, 11.9889(3), of the sample that was processed directly at 1300 °C for 2 h. Electrical conductivity was measured to be 4.0(1) S·cm^−1^ at room temperature, which is two orders of magnitude larger than the sample processed at 1300 °C for 2 h. The lattice parameter and electrical conductivity were near the same values of the sample that was processed directly at 1300 °C for 6 h. 

**Figure 10:**
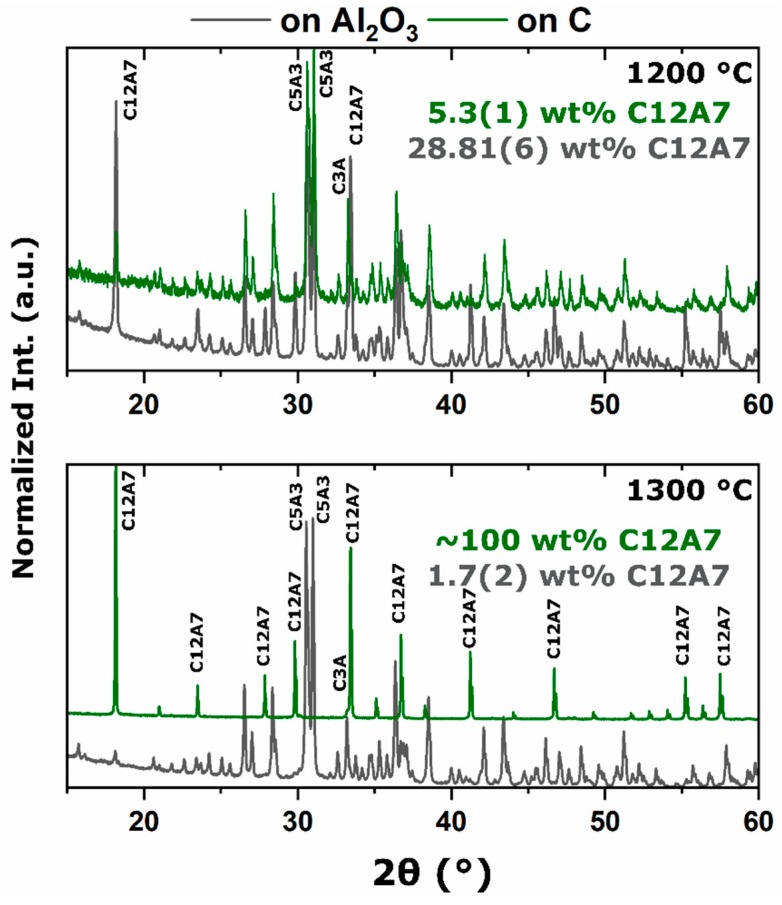
Comparison between as-synthesized oxy-C12A7 processed on Al_2_O_3_ (gray) vs carbon (green). The C5A3 and C3A decomposition pathway was observed in both scenarios.

To assess the effect of intimate contact with the carbon source, a C12A7 pellet was pressed and then placed between two alumina plates. Samples were then processed at 1200 and 1300 °C in the high vacuum furnace for 2 and 6 h, respectively. Comparison between these samples and those processed in the carbon die for equivalent process durations demonstrated a clear change in C12A7 phase content (Figure 10). At 1200 °C, five times as much C12A7 was observed when processed on Al_2_O_3_ compared to samples processed on carbon. At 1300 °C, on carbon, C12A7 phase purity was observed; however, when processed on Al_2_O_3_, only 1 wt% of C12A7 was observed; the decomposition products of C5A3 and C3A were observed. The observed lattice parameter of the sample processed on Al_2_O_3_ was that of the oxy-C12A7 structure, indicating its presence was only due to the lack of full decomposition after 6 h. 

### 3.2. Direct Electride Formation

**Figure 11:**
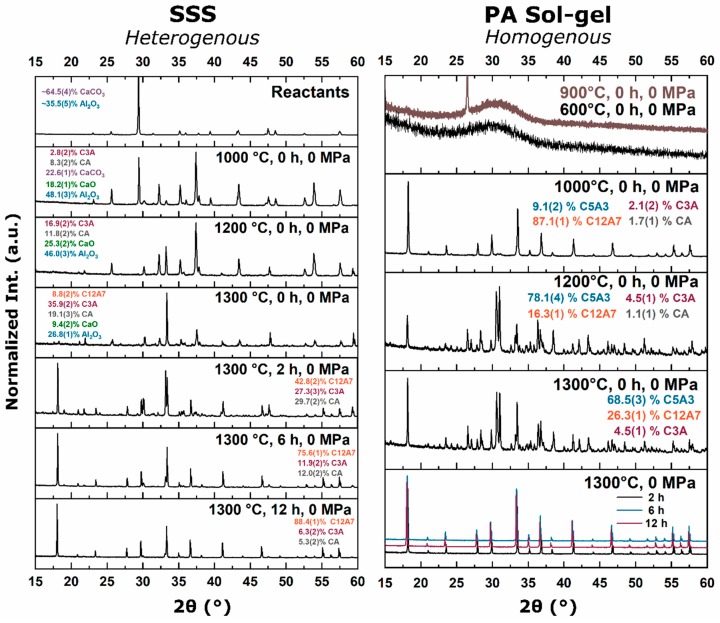
(**Left**) Kinetic phase evolution behavior of the heterogenous SSS reactants with processing temperatures of 1000, 1200, and 1300 °C and a 0 h processing time, and for processing times of 2, 6, and 12 h for samples with a processing temperature of 1300 °C. (**Right**) Kinetic phase evolution of calcined homogenous polymer assisted sol-gel reactants with processing temperatures of 600, 900, 1000, 1200, and 1300 °C and a processing time of 0 h, and for processing times of 2, 6, and 12 h for samples with a processing temperature of 1300 °C.

When the starting powder was changed from oxy-C12A7 to solid-state synthesis (heterogenous) or polymer assisted sol-gel (homogenous) reactants, formation and stability of the electride-C12A7 structure was observed once the process temperature increased to 1300 °C regardless of starting reactants (Figure 11). For heterogenous reactants, as the temperature was raised to 1000 °C, CaCO_3_ decomposition is observed. C12A7 did not form; however, the off C12A7 stoichiometry Ca rich C3A phase and Al rich CA secondary phases were observed. C3A and CA phase fractions increased as the process temperature was raised, and at 1300 °C, C12A7 phase formation was observed. As the process time was increased at 1300 °C, the observed C12A7 content increased to 88 wt% after 12 h with C3A and CA making up the remaining 12 wt%. The surface and bulk color of the consolidated sample remained white through 1300 °C for a processing time of 0 h; a process time of 0 h indicated a fast cooling once the process temperature was achieved. A gray and black color was observed as process time was increased from 2 h to above 6 h, respectively. When ground to a powder, the observed color was white through processing at 1300 °C for 2 h, after which, the color turned to a dark green for longer processing times, indicating electride formation. 

The phase evolution of the homogenous reactants had a different path than the SSS reactants resembling the process observed when reducing converting oxy-C12A7 to electride-C12A7 (Figure 11). An amorphous product was observed through 900 °C with the observed peak being characteristic of residual carbon impurities on the surface of the sample. At 1000 °C, only ≈0.25 h after the 900 °C sample due to the fast ramp rate, a white powder was characterized with C12A7 as the majority phase, indicating rapid phase formation kinetics for C12A7. As the process temperature was raised above 1050 °C, the decomposition of the C12A7 phase to C5A3 and C3A was observed. At 1300 °C, formation and stability of C12A7 was observed, and C12A7 phase purity was found after processing for 2 h at 1300 °C. Phase purity is likely to be reached earlier based on the quick kinetics observed during decomposition and formation characterized at lower temperatures. The sample color remains white through 1300 °C for a processing time of 0 h; however, as the processing time was increased, the sample color changes to black. When the consolidated samples were ground, a white powder was observed through 1300 °C for a processing time of 2 h, after which a change to green was observed as process time increased. 

Rietveld refinements were performed to quantitatively analyze the structural changes during the electride formation process. Analysis as a function of process temperature and time shows the initial room temperature lattice parameters of C12A7, crystalized in both processes at 1300 °C, to be close to 11.993Å with a relatively small increase as process time is increased (Table 1**)**.

Resistivity measurements demonstrate that the structure and color observation was correlated with a change in electrical conductivity and that direct formation of the electride-C12A7 from common reactant mixtures was observed. Direct formation of C12A7 electrides from the PVA reactant reached conductivity values of 3 and 5 S·cm^−1^ after 6 and 12 h, respectively. The sample processed at 1300 °C for 2 h was resistive with a conductivity of 4.0 × 10^−3^ S·cm^−1^. Samples synthesized from SSS reactants only achieved a high C12A7 phase concentration after a long process duration and the conductivity value after processing for 12 h at 1300 °C was 16 S·cm^−1^.

## 4. Discussion

The electride-C12A7 phase was achieved through conversion of oxy-C12A7 and through direct formation from heterogenous SSS and homogeneous sol-gel reactants. The degree of electron concentration agrees well with other reports and the structural evolution as a function of process time and temperature elucidated three key points for discussion: what is responsible for the change in phase equilibria at 1300 °C leading to C12A7 phase stability, what is the mechanism for electride formation, and what leads to the plateau in the degree of reduction? 

### 4.1. High Temperature C12A7 Stability

The thermodynamic phase equilibria changed as a function of process temperature and time. The as-synthesized oxy-C12A7 starting phase was stable up to 1000 °C followed by instability and decomposition as the process temperature was raised to 1200 °C. This decomposition is well documented in the C12A7 literature and coupled to the instability of the “free” occluded O^2−^ anion and activation of Ca diffusion above 1050 °C, leading to a change in thermodynamic equilibria [15]. This thermodynamic equilibrium was also observed from reactant mixtures. For heterogenous reactants no C12A7 formation was observed as the temperature was raised to 1200 °C with C3A and CA being favorable. Homogenous reactants led to C12A7 formation at low temperatures (≤1000 °C) due to kinetic favorability, but at higher temperatures, C12A7 decomposition was observed and C5A3 became the thermodynamic favorable phase. A shift in phase equilibria came when the process temperature was increased to 1300 °C. C12A7 phase purity was observed as processing time was increased regardless of the starting point. 

During the furnace ramp, the decomposition of previously synthesized oxy-C12A7 to C5A3 and C3A did not occur instantaneously, and as the temperature was rapidly increased to 1300 °C, at a ramp rate of 480 °C/h, full decomposition would not occur before 1300 °C. Once at 1300 °C the oxy-C12A7 structure was mostly retained but actively decomposing. The secondary phases of C3A and CA were observed, indicating a change in the decomposition kinetics brought on by higher cationic diffusion, but after 6 h, C12A7 phase purity was achieved correlated with approaching the asymptotic values of conductivity, lattice parameter, and occupied cages. This suggests that a decomposition of oxy-C12A7 and reformation back to C12A7 was fundamental to achieving the electride phase. 

This reformation process was corroborated for the oxy-C12A7 conversion process through a multi-step processing experiment. The sample was initially fired isothermally at 1200 °C for 2 h before increasing the process temperature to 1300 °C for 2 h. Full decomposition to C5A3 occurred at the lower temperature and the presence of the C12A7 structure at the higher temperature corroborated the recrystallization process. The atomic structure lattice parameter and sample conductivity were greater than that when directly processing at 1300 °C for 2 h, but slightly less than that of a sample processed at 1300 °C for 6 h. This suggested that the rate of approaching the asymptotic atomic and electron structure was not dependent on the time spent at 1300 °C, but on the total time spent at a temperature where decomposition could occur and the degree of off-stoichiometry from C12A7 of the decomposed products; the more off-stoichiometric the intermediary calcium aluminate phases, the more diffusion was necessary to form C12A7.

The exact transformation dynamics were obscured by the presence of oxy-C12A7 and the approach to the asymptotic values could be related to an average of the oxy-C12A7 and the newly formed electride-C12A7 phase. The direct formation of electride-C12A7 from reactant mixtures was not obscured by the presence of previously synthesized oxy-C12A7. The fact that there was no approach to the asymptotic values suggested that the electride forms from a direction crystallization process. Structural characterization showed that the asymptotic structural values observed in the conversion of oxy-C12A7 to electride-C12A7 were the intrinsic starting values of the electride-C12A7 formed through the direct formation process. These structural observations led to the question of “what thermodynamic change or activated kinetic process leads to this change in equilibria?” Oxy-C12A7, full electride-C12A7, or a mixed oxy/electride-C12A7 structure would all be unstable under these experimental conditions (>1050 °C in a dry reducing atmosphere) indicating that a high temperature stable anionic species must be aiding in phase stability [13,14,15]. 

As the processing temperature was raised from 1200 to 1300 °C, the formation and stability of C12A7 was observed from the three starting points. The rise in temperature was correlated with a visible color change of samples from the as-synthesized white to a black; however, when the consolidated samples were ground, the powder color was dark green; the ultimate conductivity achieved was too low for the change to black to be correlated to an increase in free Drude carriers, indicating that the change to black resulted from another reason [31]. This observation coupled with the observance of C in the microstructure indicated the increase in system energy activated the diffusion of C whose availability as an anionic species led to high temperature C12A7 stability; the diffusion of C into hot-pressed materials (similar experimental conditions, but with an addition of pressure) is well documented with the carbonaceous source of the die [32,33]. 

To evaluate this theory, the carbon source was removed and replaced with alumina and no observed stabilization occurred; phase equilibria consisted of C5A3 and C3A at 1300 °C. At 1200 °C, the kinetics of decomposition to C5A3 and C3A were sluggish compared to when the sample was directly in contact with C, indicating that the presence of sample–die interfacial carbon played a role in the instability of the oxygen anion. This correlates well with the observation that decomposition increased with a shift toward more reducing environments from a dry oxidizing, to vacuum, and ultimately to a carbonaceous vacuum environment [15,34]. The activation of carbon diffusion and phase equilibria achieved when removing the carbon source clearly indicated that a solid-state interaction of C led to the C12A7 phase. 

The structural observations from this solid-state experimentation match well with the equilibria theory proposed in characterizing electride-C12A7 synthesized from high temperature melts [17]. No inference can be made on the species of carbon, but in the solid-state process, the diffusion of carbide species in C12A7 is unlikely. The cages may act like nano-reactors forming the carbide anion, but diffusional processes likely require the disassociation and interstitial diffusion of individual carbon species. This is a similar process to that proposed for hydroxide diffusion [21]. 

### 4.2. Electride Formation Mechanism

The structural investigation provided strong experimental evidence for the necessity of C for high temperature C12A7 crystallization, but the mechanism behind electride conversion and the asymptotic behavior in electride conversion were still difficult to elucidate with a high degree of confidence. The theory for electride conversion put forth from melt formed electride-C12A7 is that the $C_{2}^{2-}$anion is responsible for C12A7 crystallization and anion instability is observed during cooling or instantaneously after nucleating the cages [17]. In this study anion instability required a diffusionless phase transformation as the temperature was quickly quenched to below 600 °C; this was further limited due to the slow dissociative diffusion process proposed for carbon diffusion. Instantaneous instability after nucleating the cages did not explain the retained phase stability of electride-C12A7 for extended time durations in a thermodynamic regime where decomposition would occur. The electron is not capable of sustaining the C12A7 framework and decomposition was observed in non-carbonaceous environments [13,14]. The second consideration is the electride conversion process, which does not continue to full electron concentration, resulting in semi-conducting behavior reported in other carbonaceous reduction studies [3,16,35]. The aforementioned model does not accurately characterize the structural behavior reported here and another model is needed to characterize the electride formation observed in this report. 

An alternative model proposed by Jiang et al. assumes that carbide anion diffusion into the C12A7 particles will not occur and that the reduction process occurs through the interfacial reaction of occluded O and microstructural carbon. Two issues that remain unresolved with this model are: (1) the formation of oxy-C12A7 at higher temperature, where it is thermodynamically unstable, and (2) the long duration of phase stability under conditions where neither oxy-C12A7 nor electride-C12A7 are thermodynamically favored. 

We propose a new theory that builds upon the two current theories where C12A7 formation occurs with mixed C and O occluded anions and the electride conversion occurs through interaction of these occluded species and interfacial carbon species. Previously, the theory that carbide was responsible for the crystallization of C12A7 from a high temperature melt assumed no other templating anions besides C were available. This is a reasonable assumption in the sense that even if there was excess oxygen in the melt, it is not thermodynamically favorable to form oxy-C12A7, but in the solid-state system, extra-framework oxygen is accounted for in the chemical equilibria. The main formation pathways observed for C12A7 formation occur through the interaction of off-stoichiometry calcium aluminate phases:

$$5{\mathrm{Ca}}_{3}{\mathrm{Al}}_{2}O_{6}\;\left(C3A\right)+9{\mathrm{CaAl}}_{2}O_{4}\left(\mathrm{CA}\right)↔2{\left[{\mathrm{Ca}}_{12}{\mathrm{Al}}_{14}O_{32}\right]}^{2+}:O^{2-}(\mathrm{oxy}-C12A7)$$

$${\mathrm{Ca}}_{3}{\mathrm{Al}}_{2}O_{6}\left(C3A\right)+9{\mathrm{Ca}}_{5}{\mathrm{Al}}_{6}O_{14}\left(C5A3\right)↔4{\left[{\mathrm{Ca}}_{12}{\mathrm{Al}}_{14}O_{32}\right]}^{2+}:O^{2-}(\mathrm{oxy}-C12A7)$$

$$12\mathrm{CaO}\left(C\right)+7{\mathrm{Al}}_{2}O_{3}\left(A\right)↔{\left[{\mathrm{Ca}}_{12}{\mathrm{Al}}_{14}O_{32}\right]}^{2+}:O^{2-}(\mathrm{oxy}-C12A7)$$

If stoichiometrically balanced utilizing the framework stoichiometry (Ca_12_Al_14_O_32_), an excess of oxygen is needed. Support for this theory is observed during the direct formation of the homogeneous sol-gel reactants where C12A7 formation occurs between 900°C and 1000 °C where carbon diffusion has yet to be activated and the energy for oxy-C12A7 thermodynamic instability has yet to be reached; the only available anion for C12A7 formation is oxygen contained within the reactants. The presence of oxygen in the second theory by Jiang is plausible with a realized oxygen source and interfacial reduction can occur; however, the stability of the oxy/electride-C12A7 structure is not explained if we assume a single anion model. 

It was previously assumed that carbide diffusion into the C12A7 particle is unlikely, but the diffusion of mono-atomic carbon species is likely. Structural analysis shows that the cage “windows” are 3.7 Å in diameter and carbon diffusion is readily observed interstitially in Fe, where nearest neighbor bond distances are <3 Å [36,37]. The idea of mixed anion stability is observed in the C12A7 phase space [15,28,36]. Under humid conditions, a mixture of oxygen- and hydroxide-occupied cages leads to phase stability above 1050 °C where oxy-C12A7 instability is observed under dry conditions [15]. With the presence of oxygen and carbon species, phase stability is understood and there is a clear electride formation process. The negative enthalpy of formation for the reaction of oxygen and C drives the formation of anion vacancies and electron injections; this may occur through the interaction of occluded species, as well as interfacial species, as proposed by Jiang et al. [19].

### 4.3. Plateau in Electride Formation

The final point for consideration is the driving factor behind the ultimate level of reduction reaching a semi-conducting state rather than the metallic type conductivity state achieved with other techniques. The driving factor behind electride conversion is thermodynamic equilibria and the presence of a negative enthalpy chemical reaction. In order to reach this thermodynamic equilibrium, the diffusion of occluded anions from the C12A7 phase to the oxygen getter needs to occur, resulting in anionic vacancies. The plateau in conductivity is either limited by the intrinsic reduction process, the kinetic diffusion of anionic species, or the thermodynamic driving factor. An example of the intrinsic reduction process limiting electron concentration is observed in the hydrogen gas reduction process [38]. However, the same equilibrium observed in this study was observed when treating oxy-C12A7 in a ${\mathrm{CO}}^{-}$-reducing atmosphere; in this case there were no carbon species occupying the cage and only $O^{2-}$, suggesting that another limiting factor should be considered [16]. 

**Figure 12:**
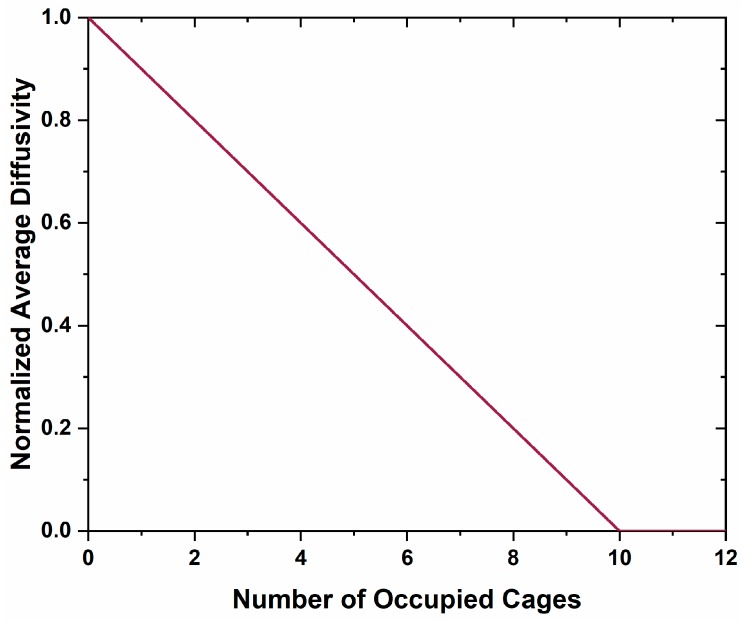
Normalized calculated average diffusivity as a function of the number of occupied cages. A linear decrease is observed as the occupation of diffusional sites increases, leading to a diffusion of zero once 83% of the cages are occupied.

The Ca metal reduction method only achieves moderate conductivity (≈100 S·cm^−1^) due to the blockage of the kinetic pathway responsible for anionic vacancy injection [39]. In the mixed oxygen and carbon occluded anion C12A7, high oxygen mobility toward the occluded carbon species would be observed. The formation of CO would take place inside of the cage, similar to the hydration of C12A7 and hydroxide diffusion, and once the new polyatomic anion has formed, its diffusion and mobility will be dramatically hindered relative to monoatomic species; a diffusion method similar to $OH^{-}$ diffusion may occur with the dissociation of CO, interstitial diffusion of C, and then recombination in the adjacent cages [21]. The large diatomic anion will act as a blocking agent for future oxygen, carbon, and electron diffusion, limiting further formation of anion vacancies and limiting mobility of localized carriers. There is a significant difference between the blocked kinetic pathways in the Ca metal reduction method versus this method. In the former the kinetic pathway blockage is due to a layer of CaO encasing the material, where this discussion suggests a blocking of diffusion pathways by molecules within the cages. This is unlikely due to the high interconnectivity of the cages, where each cage has 12 nearest neighbor cages. Application of a percolation theory, Equations (1) and (2), of a system with 12 neighbor connectivity demonstrates that while diffusion would decrease with an increase in the number of occupied cages, it would not cease completely until 10 cages are occupied (Figure 12) [40].

(1) $$\frac{D_{m}}{D_{0}}=\frac{1}{2}\left\{A+{\left[A^{2}+\frac{4f}{\left(\left(\frac{z}{2}\right)-1\right)}\right]}^{\frac{1}{2}}\right\}$$

(2) $$A=1-p+fp-\frac{f+p-fp}{\left(\frac{z}{2}-1\right)}$$

where $\frac{D_{m}}{D_{0}}$ is the normalized average diffusivity; p is the fraction of blocked cages; *f* is the diffusivity through blocked pathways, which was set to zero; and *z* is the average coordination number to nearest neighbor diffusion sites, 12 in this case [40]. The Fourier difference maps demonstrate a decrease in occluded position scattering density corresponding to cage occupancy, making the equilibria observed unlikely due to a blocking of diffusion kinetic pathways in the cage structure. Therefore, another process should be considered.

**Figure 13:**
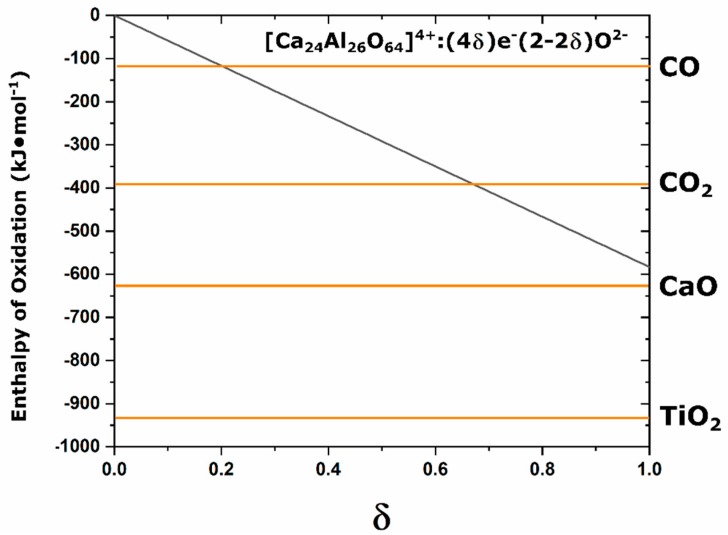
Enthalpy of oxidation of C12A7 as a function of electron conversion (δ) compared to the enthalpies of oxidation of C, Ca, and Ti [16,41,42,43].

The reduction of C12A7, in all techniques, is driven by the negative enthalpy of oxidation of an external chemical species. Oxidation of Ca and Ti have large negative enthalpies of formation, −635 and −940 kJ·mol^−1^, respectively [41,42]. The enthalpy of formation for CO and CO_2_ is relatively lower at −110.53 and −393.52 kJ·mol^−1^, respectively [41]. While C12A7 is an oxidizing agent with oxygen in the cage, the full electride has a positive enthalpy of formation, indicating that the full oxygen occupation is thermodynamically preferred and oxidation of the fully converted electride has an enthalpy of formation between −425 and −600 kJ/mol [16,43]. This makes the electride a reducing agent (the ability for C12A7 to be both an oxidizing and a reducing agent as a function of processing is part of what leads to the observed high functionality). Figure 13 compares the enthalpy of oxidation as a function of electride conversion with the enthalpy of oxidation for C, Ca, and Ti superimposed; the enthalpy of oxidation for electride-C12A7 decreases as the degree of electron concentration decreases and the slope of this line is −580 kJ·mol^−1^ [43]. This representation assumes that these values are comparable at elevated temperatures, that the enthalpy of formation from elemental species is a good approximation for that observed in the complex C12A7 system, and that the linear decrease in enthalpy as a function of electron concentration is valid under the carbonaceous vacuum conditions. TiO_2_ and CaO have enthalpies of formation with magnitudes higher than that of the full electride, indicating that oxidation of these cations will occur in favor of oxidation of the electride-C12A7 with the highest possible electron occupation. However, for the oxidation of C, both the oxidation to CO_2_ and CO have lower enthalpies of formation with magnitudes less than that of the full electride. This makes the formed electride-C12A7 a competing reduction agent and equilibrium will occur where the enthalpy of oxidation of electride-C12A7 is equal to the enthalpy of oxidation for C. At this point, the electride conversion process will cease and an equilibrium will be observed irrespective of further processing. Based on our structural observations, this coincides with one cage occupied and an electrical conductivity of ≈15 S·cm^−1^; the exact ratio of O:C species and valence of these remaining species is unable to be determined in this study. The presence of the remaining C species leads to the high temperature stability at the electride-conversion equilibrium.

This model is supported by the observed structural characterization. However, future work is needed to fully identify the type and presence of occluded C species. Such a task is challenging due to the occluded position disorder, low occupation, and low Z of the anions of interest. The kinetic processes during reduction involve changes in local short-range order while retaining the long-range order of the framework. In situ diffraction techniques as a function of temperature, atmosphere, and time, probing both local and long-range order, could provide the experimental verification of the proposed reduction methods with higher resolution than the limited process data points able to be analyzed in ex situ techniques. Atomistic modelling can be employed by utilizing reactive force field potentials to determine thermodynamic properties and confirm the nano-reactor theory responsible for electride C12A7 formation. 

## 5. Conclusions

Successful electride-C12A7 formation was observed in a high vacuum carbonaceous environment, not only from the conversion of pre-synthesized oxy-C12A7, but also the direct formation through heterogenous solid-state and homogeneous non-carbonaceous polymer assisted sol-gel reactants. This is the first report of direct electride formation from CaCO_3_ + Al_2_O_3_ and a non-carbonaceous sol-gel reactant. The following conclusions can be made:

1. Direct contact with a carbon source, at 1300 °C to activate carbon diffusion, and decomposition of the oxy-C12A7 phase, is suggested for the reformation to a new thermodynamically favorable phase in a dry reducing condition. The presence of other calcium aluminate compounds preserves the oxygen availability needed to form oxygen-occupied C12A7 and the incorporation of C as an occluded anion leads to a mixed occupancy high temperature phase stability much like the incorporation of OH^−^ does in high temperature humid environments.
2. The reduction method is proposed to be an interaction between the occluded C and O anions as well as interfacial C and O with an observed plateau in atomic structural parameters and electronic conductivity, regardless of extended processing time. This represents a new electride formation model.
3. The most plausible theory for the plateau in electrical conductivity involves the competing thermodynamics of oxidation where the reducing power of the electride-C12A7 phase and the C species become equal in strength.

Further experimentation combining in situ and total scattering diffraction, as well as atomistic modeling, is necessary for further validation and clarification of anionic species ratios, reduction thermodynamic information, and short-range order in C12A7. This presents a shift in theory behind the carbonaceous C12A7 electride formation derived from a structural characterization of the electride-C12A7 formation.

## Figures and Tables

**Table 1:** Comparison of C12A7 lattice parameter after undergoing processing with a carbonaceous die in a vacuum furnace from a variety of starting points; previously synthesized oxy-C12A7, heterogenous solid-state reactant mixture, and atomic homogenous calcined polymer assisted sol-gel reactant mixture are the various starting points. Errors are represented as 3σ.

Temp. (°C)	Time (h)	Solid-State Reaction		Wet-Chemistry
		Synth. C12A7	Heterogenous SSS Reactants (Al_2_O_3_ + CaCO_3_)	Homogenous PA Sol-gel Reactants (Calcined 600 °C)
		*a* (Å)	*a* (Å)	*a* (Å)
**As-Synth.**	-	11.9808(2)	-	-
**1000**	2**, 0*	11.9849(2)**	-	11.9936(3)*
**1300**	**0**	-	11.992(1)	11.9920(3)
	**2**	11.9889(2)	11.9923(3)	11.9918(3)
	**6**	11.9915(2)	11.9933(2)	11.9925(3)
	**12**	11.9924(1)	11.9937(3)	11.9928(2)
	**72**	11.9933(2)	-	-

*,** Asterix are used to differentiate between processing time at 1000 °C for synthesized C12A7 (**2 h) and homogenous sol-gel reactants (*0 h).

## Supplementary Materials

The following are available online at http://www.mdpi.com/1996-1944/12/1/84/s1, Figure S1: XRD patterns of HUP-processed oxy-C12A7 comparing the final characteristic structure after sintering at 1300 °C for 2 h. The black process involved an additional step at 1200 °C for 2 h prior to the 1300 °C for 2 h. This was to allow for full decomposition of the C12A7 structure at 1200 °C where oxy-C12A7 is unstable. Subtle changes in characteristic peaks were observed, indicating that both samples were primarily C12A7; however, when no hold at 1200 °C was implemented, an increase in CA characteristic peaks at 30° 2Θ was observed. Structural Rietveld refinement, detailed in the main text, indicates that a structural difference between the two samples is a significant indication that the electride formation rate was increased when the hold at 1200 °C was observed.
