# Supplementary material for: Direct Formation and Structural Characterization of Electride C12A7
Source: Materials (Basel). 2018 Dec 27;12(1):84. doi: 10.3390/ma12010084 (PMC6337680; doi:10.3390/ma12010084)
Supplement: Supplementary file 1 [file materials-12-00084-s001.pdf]

Direct Formation and Structural Characterization of Electride C12A7

J.R. Salasin [1,2], S.E.A Schwerzler [1], R. Mukherjee [3], D.J Keffer [1], K.E. Sickafus [1], and C.J. Rawn [1,2,*]

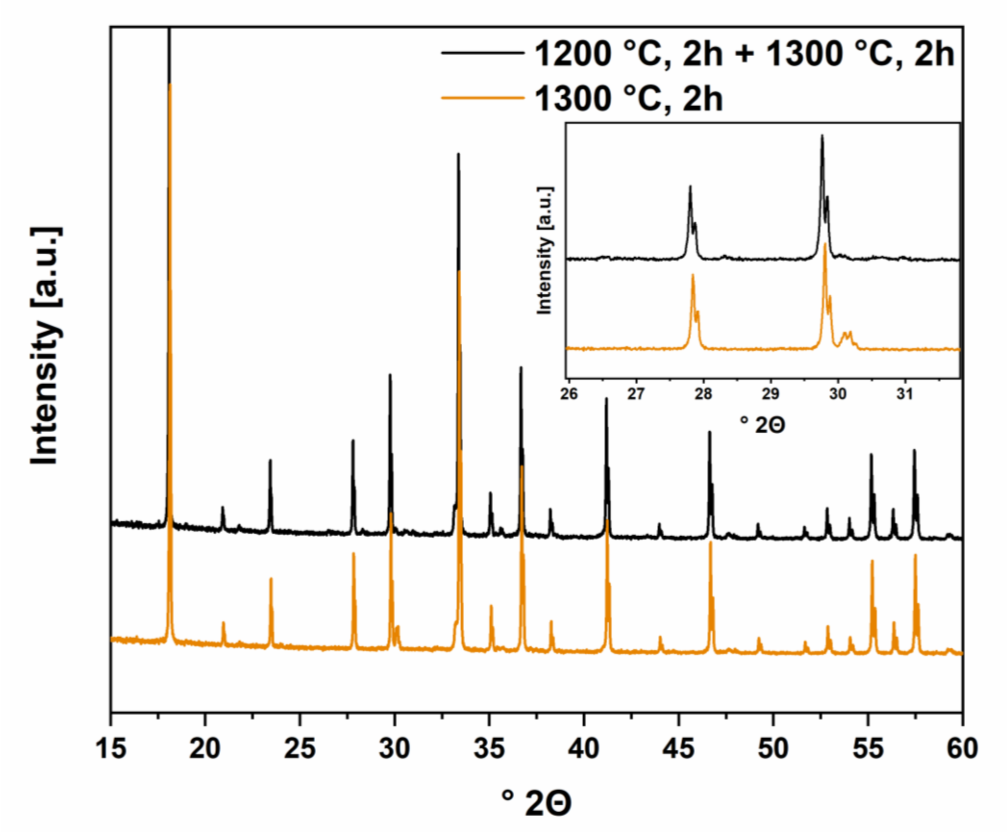

Figure S1. XRD patterns of HUP-processed oxy-C12A7 comparing the final characteristic structure after sintering at 1300 °C for 2 h. The black process involved an additional step at 1200 °C for 2 h prior to the 1300 °C for 2 h. This was to allow for full decomposition of the C12A7 structure at 1200 °C where oxy-C12A7 is unstable. Subtle changes in characteristic peaks were observed, indicating that both samples were primarily C12A7; however, when no hold at 1200 °C was implemented, an increase in CA characteristic peaks at 30° 2Θ was observed. Structural Rietveld refinement, detailed in the main text, indicates that a structural difference between the two samples is a significant indication that the electride formation rate was increased when the hold at 1200 °C was observed.
